# Optimising Assessments of the Epidemiological Impact in the Netherlands of Paediatric Immunisation with 13-Valent Pneumococcal Conjugate Vaccine Using Dynamic Transmission Modelling

**DOI:** 10.1371/journal.pone.0089415

**Published:** 2014-04-02

**Authors:** Elisabetta De Cao, Alessia Melegaro, Rogier Klok, Maarten Postma

**Affiliations:** 1 Department of Pharmacy, University of Groningen, Groningen, Netherlands; 2 Department of Economics, Econometrics and Finance, University of Groningen, Groningen, Netherlands; 3 Policy Analysis and Public Management Department and Dondena Centre for Research on Social Dynamics, Bocconi University, Milan, Italy; 4 Pfizer bv, Specialty Care Business Unit, Capelle a/d IJssel, Netherlands; University of Malaya, Malaysia

## Abstract

This work is the first attempt to quantify the overall effects of a 13-valent pneumococcal conjugate vaccine (PCV13) vaccination programme in the Dutch population taking into account all the direct and indirect effects of the vaccine on invasive pneumococcal disease. Using available Dutch data, a dynamic transmission model for the spread of pneumococci and potential subsequent invasive pneumococcal disease has been adapted to the Dutch setting. Overall, invasive pneumococcal disease cases in the Netherlands are predicted to decrease from a pre-vaccination level of 2623 cases annually to 2475, 2289, 2185, 2179, and 2178 cases annually 5-, 10-, 20-, 30-, and 40-years, respectively, post-vaccination. Therefore, vaccination with PCV13 in the Netherlands is predicted to lower invasive pneumococcal disease cases per year by up to 445 cases in the medium- to long-term. The results are quite robust for the sensitivity analyses performed on the parameters that regulate herd immunity and competition between vaccine and non-vaccine types.

## Introduction

*Streptococcus pneumoniae* (pneumococcus) is the main cause of pneumonia, acute otitis media, bloodstream infections (bacteraemia) and severe forms of meningitis [1]. The burden of disease related to the bacterium *S. pneumoniae* is highly relevant from a public health perspective [2]–[4]. In nature, >90 immunologically different pneumococcal serotypes have been identified, which differ in the chemical compositions of their polysaccharide capsules. Each serotype behaves differently in respect to several properties, including invasiveness, affinity towards age groups, disease manifestation, carriage, and outbreaks. Preventing pneumococcal disease caused by specific serotypes is possible through vaccination.

The 7-valent pneumococcal conjugate vaccine (PCV7), which became available in 2000, contains serotypes 4, 6B, 9V, 14, 18C, 19F, and 23F. The 10-valent (PCV10) and 13-valent (PCV13) pneumococcal conjugate vaccines (PCVs) subsequently became available in 2009 and 2010, respectively. PCV10 includes the serotypes contained in PCV7 and three additional serotypes: serotypes 1, 5, and 7A. PCV13 contains the serotypes of PCV10 and another three additional serotypes: serotypes 3, 6A, and 19A. Conjugated vaccines administered to paediatric populations have provided direct protection in many settings [5], [6].

An additional benefit of PCV7 and PCV13 paediatric programs has been the reduction of pneumococcal disease in unvaccinated cohorts due to herd protective effects [7]–[13]. Among vaccinated children, reduction of nasopharyngeal carriage and the subsequent transmission of vaccine serotypes [14]–[19] as well as disease caused by vaccine serotypes [20], [21] has been observed. Evidence has been published on pneumococcal disease reduction in non-vaccinated populations in the US [22], UK [8], Australia [23], Netherlands [11], and other countries. Whereas herd protection would generally be expected in populations with sufficient paediatric vaccination uptake, it is in fact difficult to predict the exact epidemiological effect of broad scale use of PCVs in new settings [24], due to variation in the circulating serotypes and the inherent differences among them, as well as programmatic differences including the level of uptake and intensity of catch-up programs. Furthermore, although overall invasive pneumococcal disease (IPD) rates decreased after the introduction of PCV7, there has simultaneously been an increase of cases due to non-PCV7-vaccine serotypes. Many clinical studies have shown that, at the level of the nasopharynx, the use of PCV7 is followed by a shift from colonisation with vaccine types (VTs) to predominantly non-vaccine types (NVTs) [25]. Recent research mitigates the notion that serotype replacement would be of a scale potentially offsetting the direct and herd protection effects of the PCV. In particular, Miller *et al* (2011) [8] claim that further reductions might be achievable by use of higher-valent vaccines.

This being said, however, a great deal of uncertainty remains regarding major drivers such as long-term replacement disease and herd protection among unvaccinated individuals. Understanding the epidemiologic shifts following the introduction of PCV7 is relevant for the introduction of the newer higher-valent vaccines. Multiple factors affect serotype patterns. The vaccine could be the cause of replacement, but antibiotic use and resistance, as well as other biological characteristics of the individual serotypes, which indeed present considerable variations, may also influence serotype distributions. Previous epidemiological and health-economic analyses of higher-valent PCVs exclusively utilised static models [26]. However, currently it is well recognised that to model the changes in pneumococcal epidemiology after vaccination a dynamic model is required, which could take into account all these indirect effects. Only recently, van Hoek *et al* (2012) [27] evaluated the cost-effectiveness of the introduction of PCV13 in England and Wales using a deterministic transmission model to generate epidemiological projections of vaccine impacts. The importance of using dynamic designs, as opposed to static approaches, to explicitly model the spread of bacteria and viruses has previously been illustrated [28]–[30]. The use of different modelling approaches has shown huge differences when analysing screening for *Chlamydia trachomatis* [28] and pertussis vaccination strategies [29], [30]. To fully capture the value of infant PCV programs, it is necessary to develop a dynamic model that incorporates such indirect effects. The development of such models fits into the priority of designing innovations in health technology assessments, but requires input from mathematical, medical, epidemiological, and immunological fields, and scientific applications of such rigorous undertakings are few.

Melegaro *et al* (2010) [31] were the first to develop an age-structured transmission dynamic model that retrospectively studied the impact of PCV7 on IPD considering serotype replacement. A Susceptible-Infected-Susceptible (SIS) model was developed that considered reinfection and co-infection with NVTs and VTs. Carriage prevalence data and surveillance of IPD came from England and Wales, while degree and duration of vaccine protection and competition were derived using available US data. This study found that PCV7 vaccination could eradicate VTs, but increased the NVTs with the consequent possible increase of IPD. The authors claimed, however, that their results were sensitive to changes in protection effects and level of competition.

In the UK, a more rapid replacement of PCV7 IPD cases by non-PCV7 IPD cases was observed compared with the US. Consequently, in recent work by Choi *et al* (2011) [32], the model of Melagaro *et al* (2010) [31] was re-parameterised using vaccine coverage and IPD data from England and Wales, as well as European Union social contact mixing patterns [33]. The authors concluded that PCV7 vaccination could result in a decrease in overall IPD, mostly in children, even with a rapid replacement by NVTs within 5 years of vaccine introduction at high coverage. They also found that stopping vaccination could result in a resurgence of VT disease to pre-vaccination levels.

In the Netherlands, vaccination with PCV7 was introduced for all infants born after March 31, 2006. The schedule of vaccinations was 3 infant doses and 1 toddler dose (i.e. 3+1) at ages 2, 3, 4, and 11 months, with no catch-up campaign. As of 2011, PCV10 replaced PCV7 in the infant pneumococcal vaccination schedule. Rozenbaum *et al* (2010) [34] have found, using a static model, that the Dutch infant vaccination program of 4 doses of PCV7 is no longer cost-effective because of increases in IPD caused by NVTs. They claim that PCV13 could provide better benefits because of an increase in herd immunity and less replacement disease. The aim of this paper is to evaluate the potential effectiveness of PCV13 on IPD in a Dutch infant national immunisation program setting, using a dynamic model that takes into account disease transmission dynamics.

## Methods

The age-structured transmission dynamic model was adapted from Melegaro *et al* (2010) [31], in order to evaluate the effectiveness of PCV13 in the Netherlands. Similarly to Melegaro *et al* (2010) [31], the model here is composed of two parts, a pre-vaccination model and a transmission dynamic model, to explore the effects of the vaccination program.

### Part I: Pre-Vaccination Model

**Figure 1:**
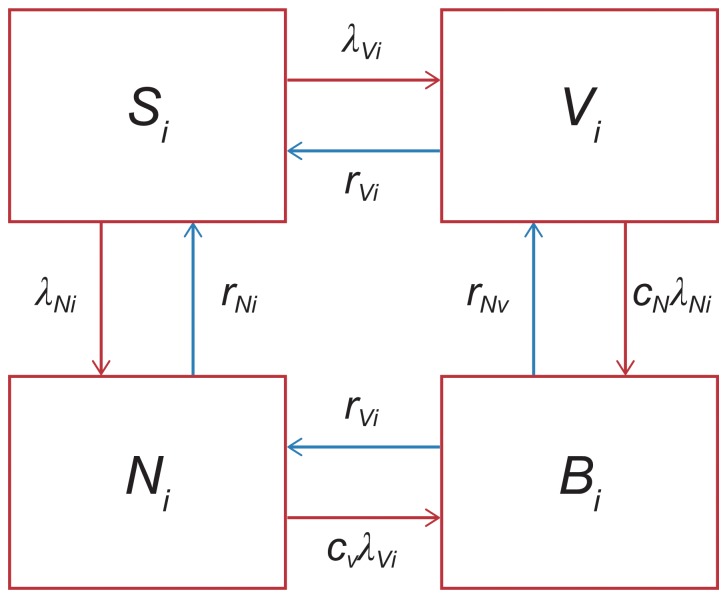
Susceptible-Infected-Susceptible (SIS) model structure. SIS model structure for estimating the forces of infection for vaccine types and non-vaccine types. Abbreviations: λ_N_, age-dependent forces of infection for non-vaccine types; λ_V_, age-dependent forces of infection for vaccine types; B, both vaccine types and non-vaccine types co-colonised; c_N_, competition parameters that determined the relative risk of acquiring non-vaccine types if already colonised by vaccine types; c_V_, competition parameters that determined the relative risk of acquiring vaccine types if already colonised by non-vaccine types; i, age group; N, non-vaccine types carriers; r_N_, recovery rate for non-vaccine types; r_V_, recovery rate for vaccine types; S, susceptible; V, vaccine type carriers;.

The forces of infection for VT and NVT were first estimated from the pre-PCV carriage data (λ_Vi_, λ_Ni_), with *i* reflecting age groups, V the VT, and N the NVT. The flowchart in **Figure 1** represents the SIS structure used to consider pneumococcal carriage. Briefly, the characteristics of the SIS model were:

- 4 compartments: S =  susceptible; V =  VT carriers; N =  NVT carriers; and B =  both VT and NVT co-colonised
- Age-dependent forces of infection for VT and NVT (λ_Vi_, λ_Ni_)
- Competition parameters that determined the relative risk of acquiring VT (NVT) if already colonised by NVT (VT) (c_V_, c_N_)
- Recovery rates for VT and NVT

The forces of infection were assumed as step functions, with a constant value in each of the following age groups: 0–1, 2–4, 5–9, 10–19, 20–29, and ≥30 years. The recovery rate was assumed to be equal to the inverse of the duration of carriage as in Cauchemez *et al* (2006) [35] and Melegaro *et al* (2007) [36]. Competition parameters were based on point estimates and lower and upper bounds of confidence intervals derived in Melegaro *et al* (2007) [36] and Choi *et al* (2011) [32]. In particular, c_V_ was fixed at 0.5, whereas c_N_ took the value 0.04 [32]. Different values of c_N_ (0 and 0.37) have been used and are reported in the sensitivity analysis, as described below. A low value of c_N_ indicates strong competition between the VT and the NVT group, i.e. carriers of one type are less likely to acquire the other type compared with susceptible individuals. The recovery rates were assumed age-dependent but equal among the two groups (r_Vi_ = r_Ni_).

A multinomial model was used to fit force-of-infection parameters to carriage data. The co-colonised individuals were grouped within the VT compartment because they were assumed to be detected as VT carriers. The multinomial model is as follows:
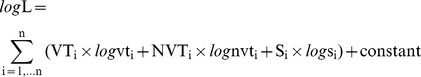
where i =  age group, VT =  observed number of VT carriers, vt =  predicted prevalence of VT carriers, NVT =  observed number of NVT carriers, nvt =  predicted prevalence of NVT carriers, S =  observed number of susceptibles, and s =  predicted prevalence of susceptibles.

The model was solved for the combination of parameter values that produced the best fit.

### Part II: Dynamic Model to Evaluate Vaccination Policies

**Figure 2:**
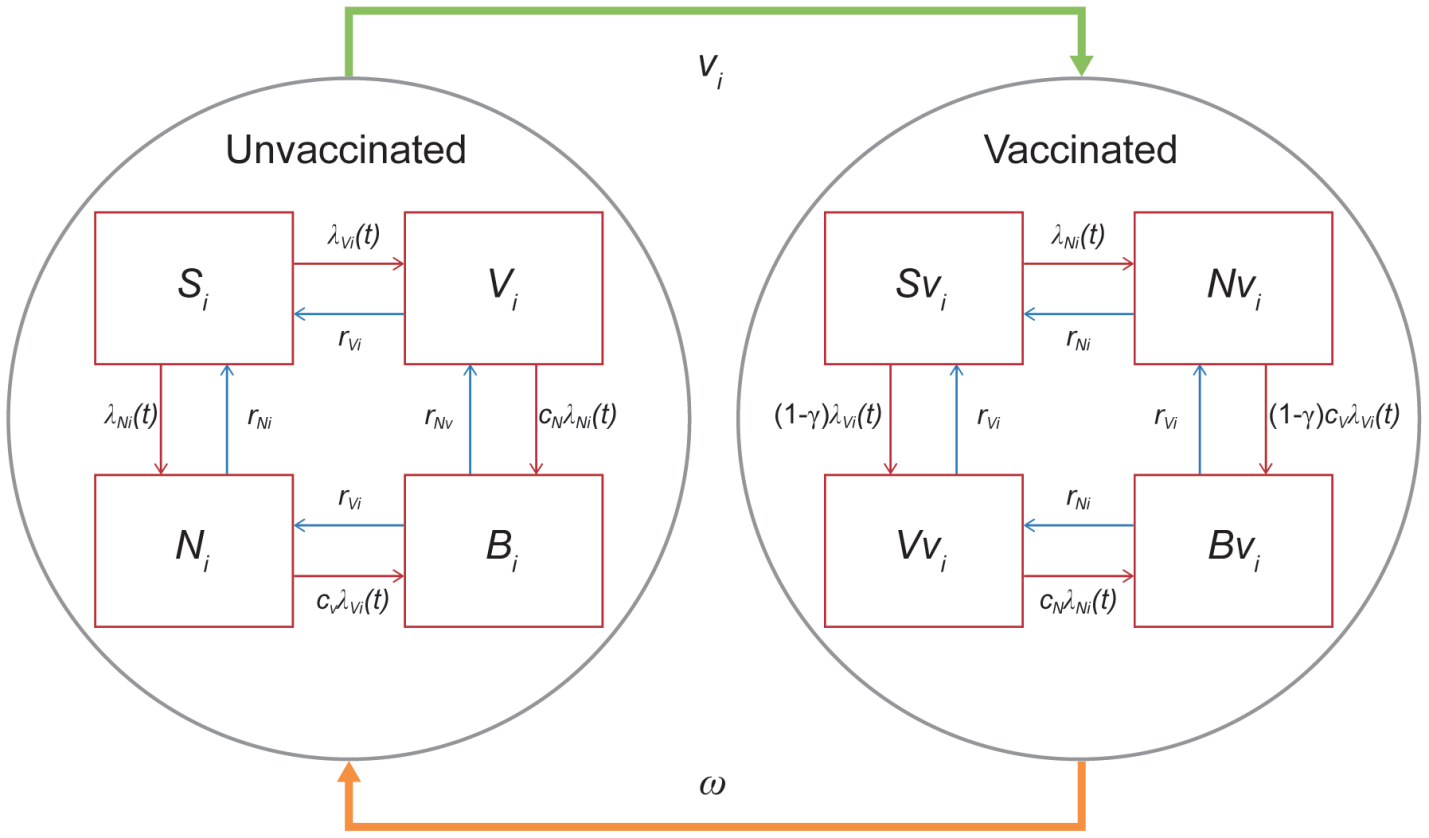
Dynamic transmission model. Flowchart representing the dynamic mathematical model used for the transmission. Abbreviations:γ_i_, degree of protection; λ_N_, age-dependent forces of infection for non-vaccine types; λ_V_, age-dependent forces of infection for vaccine types; ωi, the waning rate; B, both vaccine types and non-vaccine types co-colonised; Bv, both vaccine types and non-vaccine types co-colonised with vaccination; c_N_, competition parameters that determined the relative risk of acquiring non-vaccine types if already colonised by vaccine types; c_V_, competition parameters that determined the relative risk of acquiring vaccine types if already colonised by non-vaccine types; i, age group; N, non-vaccine types carriers; Nv, non-vaccine types carriers with vaccination; r_N_, recovery rate for non-vaccine types; r_V_, recovery rate for vaccine types; S, susceptible; Sv, susceptible with vaccination; v, vaccination coverage; V, vaccine type carriers; Vv, vaccine types carriers with vaccination.

The second part of the model consisted of a dynamic model for *S. pneumoniae*, comprising a set of ordinary differential equations (see Melegaro et al [2010] for details [31]). The flow chart in **Figure 2** shows the natural history of carriage with and without vaccination. The crucial characteristics of the model are as follows:

- The model was age-structured with 100 cohorts of individuals that age every year
- Each individual was born at age 0 and was susceptible
- The model comprised 4 compartments without vaccination: S =  susceptible; V =  VT carriers; N =  NVT carriers; and B =  both VT and NVT co-colonised
- The model comprised 4 compartments with vaccination: Sv =  susceptible; Vv =  VT carriers; Nv =  NVT carriers; and Bv =  both VT and NVT co-colonised
- Age-dependent forces of infection for VT and NVT (λ_Vi_, λ_Ni_)
- Competition parameters that determined the relative risk of acquiring VT (NVT) if already colonised by NVT (VT) (c_V_, c_N_)
- Recovery rates for VT and NVT were assumed to be equal (r_Vi_ = r_Ni_)
- Vaccination coverage was reflected by v_i_, the degree of protection by γ_i_, and the waning rate by ωi

The model was parameterised as reported in **Table 1** [32], [35]–[40], where the initial conditions were the pre-vaccination carriage prevalences for VT and NVT as estimated in Part I. The mixing parameter (ε) was estimated by fitting the transmission dynamic model to the age-specific IPD incidence rates pre- and post-PCV7 introduction, by minimising the Poisson deviance (data not shown). We assumed that the pre-PCV13 vaccination years corresponded with the pre-PCV7 vaccination years; therefore, we used carriage data estimated by van Gils *et al* (2009) [38] and an average of the IPD cases in the 2-years pre-PCV7 (June 2004–June 2006), taking the 13 serotypes included in PCV13 explicitly into account.

**Table 1:** Model parameters and ranges for the analysis of PCV13 in the Netherlands.

Parameters	Definition	Values	Ranges in sensitivity analysis	Sources [Ref.]
υ	Coverage	95%		RIVM [37]
c_N_	Competition VT	0.04	0–0.37	Choi *et al* (2011) [32]
c_V_	Competition NVT	0.50		Melegaro *et al* (2007) [36]
ε	Assortativeness	0.85	0.50–1.00	Estimated using pre- and post-PCV7 data [38], [39]
γ	Degree of protection	0.52^a^		Choi *et al* (2012) [40]
δ	Duration of protection	5 years		Choi *et al* (2012) [40]
r_vi_	Recovery rate for VT	1/duration carriage		Cauchemez *et al* (2006) [35] Melegaro *et al* (2007) [36]
r_ni_	Recovery rate for NVT	1/duration carriage		Cauchemez *et al* (2006) [35] Melegaro *et al* (2007) [36]

a We used the same degree of protection for PCV13 that was estimated by Choi *et al* (2011) [32] for PCV7. Sensitivity analysis was done on this value.

Abbreviations: NVT, non-vaccine types; PCV7, 7-valent pneumococcal conjugate vaccine; PCV13, 13-valent pneumococcal conjugate vaccine; RIVM, National Institute for Public Health and the Environment, Ministry of Health, Welfare and Sport; VT, vaccine types.

### Inputs

#### Vaccination program

The model considered routine vaccination with PCV13 of infants at 2, 3, 4, and 11 months of age (3+1), without considering any catch-up campaign.

#### Carriage prevalence

Pre-vaccination nasopharyngeal carriage rates of *S. pneumoniae* were derived from a longitudinal, randomised controlled trial implemented in the Netherlands from July 2005 and February 2006 (ClinicalTrials.gov identifier: NCT00189020). In this trial, 1003 healthy newborns and 1 of their parents were enrolled to examine the effects of a 2-dose and 2+1 dose schedule with PCV7 [38]; serotype information data were also reported in Spijkerman *et al* (2011) [39]. As data were not available for all age groups, a quadratic function was used to predict pre-vaccination carriage prevalence for missing age groups.

The above data were also used, together with recent data from a cross-sectional observational study by Spijkerman *et al* (2011) [39] on pneumococcal carriage in the Netherlands 3-years post-PCV7, to estimate the level of assortativeness in the mixing patterns.

#### Disease incidence

Infections that progressed into IPD were estimated based on fitting a model to the age-specific incidence of disease attributable to pneumococcal invasive infection as reported by the Netherlands Reference Laboratory for Bacterial Meningitis from 2004–2010, assuming a constant case-carrier ratio over time [40].

#### Population data

Model outcomes on IPD-cases were extrapolated to national totals using Dutch demographic data for the year 2006 from the Dutch Central Bureau of Statistics (Voorburg, Netherlands).

### Sensitivity Analysis

Sensitivity analyses were conducted around the competition and mixing parameters. Specifically, we tested scenarios where c_N_ was equal to 0 or 0.37, representing lower and higher competition, respectively [32]. A higher c_N_ implies a lower replacement of VT by NVT after the introduction of the vaccine. In the base-case the mixing parameter (ε) was estimated at 0.85 using PCV7 data. Subsequently we tested values of ε at 0.50 or 1.00, representing different levels of assortativeness in the mixing patterns (when ε = 1.00, mixing is totally assortative). When a more proportionate mixing pattern (ε = 0.50) was considered, it simulated a situation where additional indirect effects among unvaccinated age groups might occur.

## Results

**Figure 3:**
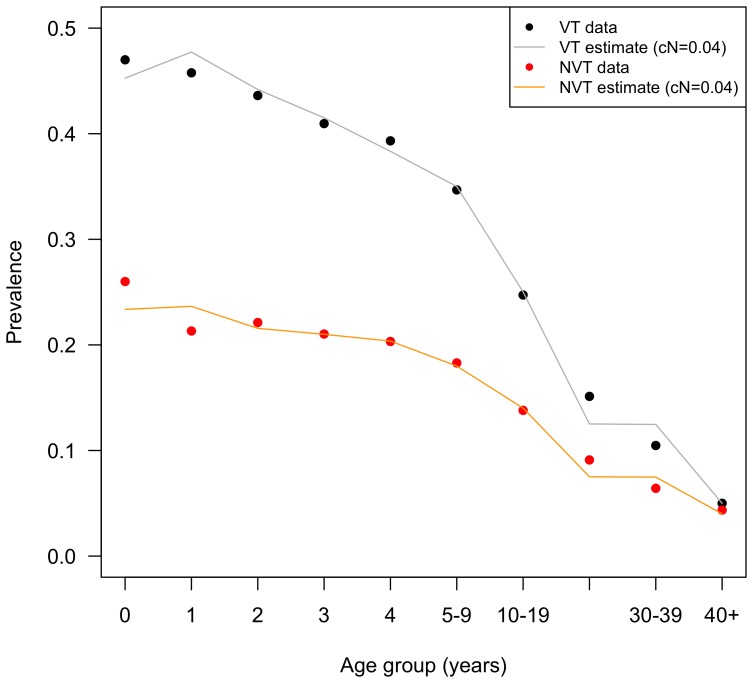
Carriage pre-PCV13. Estimated and observed carriage pre-PCV13. Prevalence rates are reported per each age or age group. Note that only the data points in Figure 3 at ages 1 and 2 years, and age group 30–39 years correspond to observed carriage data; other points were interpolated. Abbreviations: c_N_, competition parameter; NVT, non-vaccine types; PCV13, 13-valent pneumococcal conjugate vaccine; VT, vaccine types.

The pre-vaccination model resulted in a good fit to the data with the exception of ages 0 and 1 years. **Figure 3** compares observed versus estimated steady-state carriage pre-PCV13 where c_N_ = 0.04.

**Figure 4:**
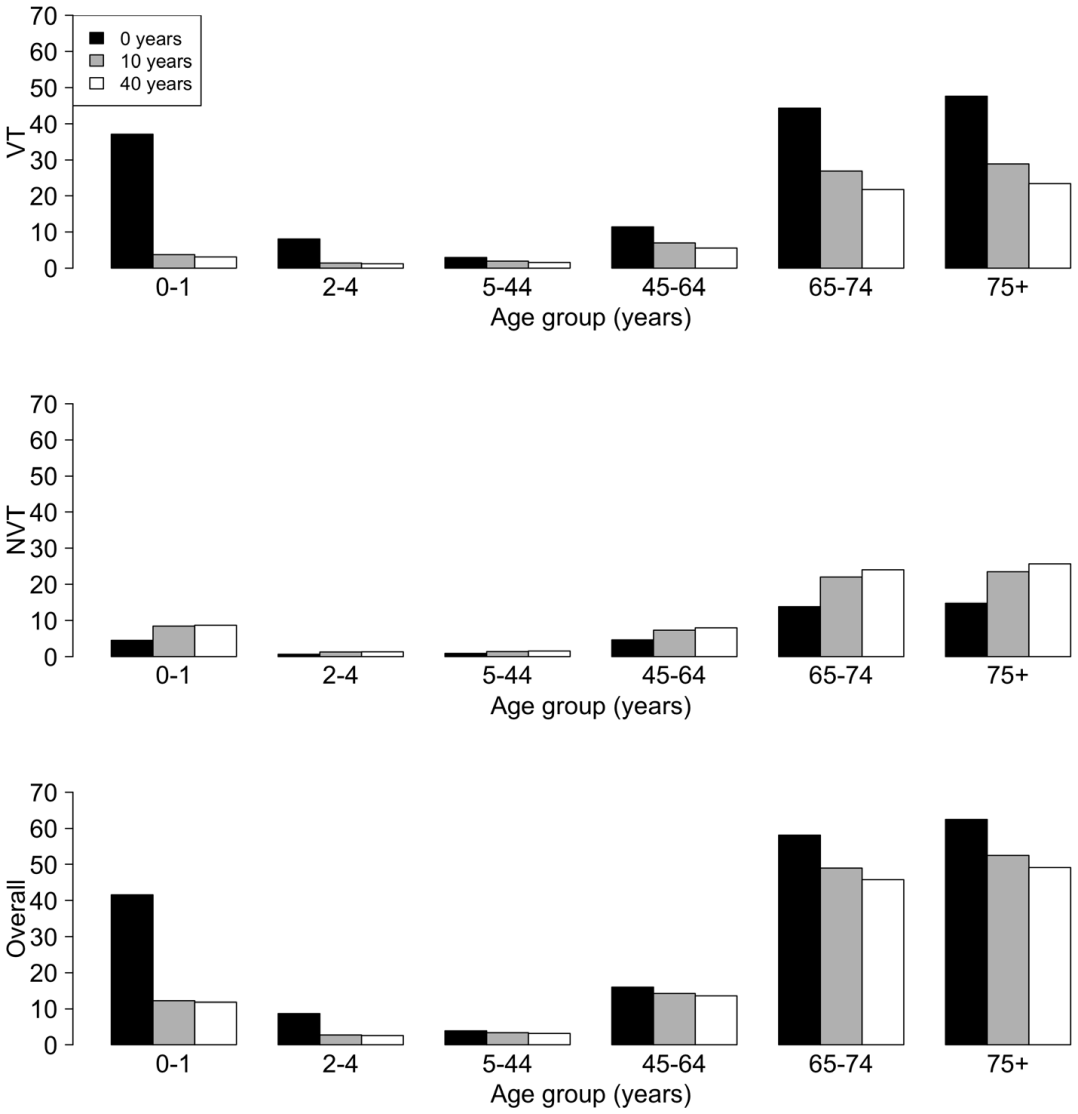
Yearly incidence of IPD. IPD yearly incidence per 100,000 population pre-vaccination (0 years) and after 10 and 40 years post-PCV13 with the competition parameter c_N_ = 0.04. Abbreviations: c_N_, competition parameter; IPD, invasive pneumococcal disease; NVT, non-vaccine types; PCV13, 13-valent pneumococcal conjugate vaccine; VT, vaccine types.

The base-case pre-vaccination, and 10 and 40 years post-PCV13 introduction IPD incidence per 100,000 population for VT, NVT, and overall IPD incidences (VT+NVT diseases) are presented in **Figure 4**. The model predicted a rapid decrease of the VT and overall incidence for the age-groups 0–1 years, and also a relevant decrease of the VT and overall incidence for the ≥65 years age groups.

**Figure 5:**
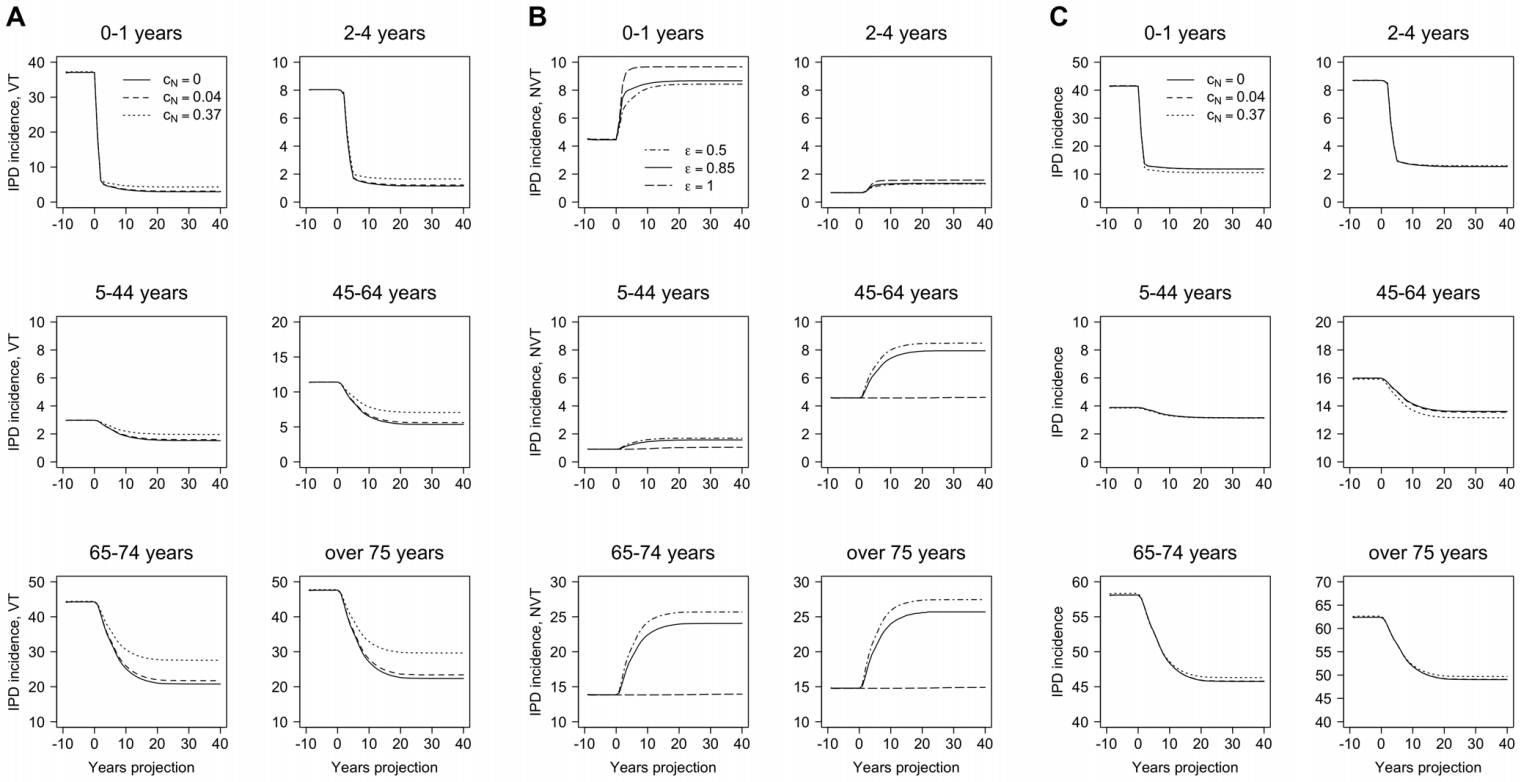
Yearly incidence of IPD with different competition parameters. **A**, IPD incidence, VT; **B**, IPD incidence, NVT; and **C**, IPD overall incidence. Yearly incidence per 100,000 population over time when different competition parameters are used: c_N_ = 0, 0.04, and 0.37; and the mixing parameter is ε = 0.85. Each panel corresponds to a different age group. Note: the y-axis scales are different on each graph. Abbreviations: c_N_, competition parameter; ε, mixing parameter; IPD, invasive pneumococcal disease; NVT, non-vaccine types; VT, vaccine types.

**Figure 5** presents VT, NVT, and overall IPD incidence per 100,000 population, over time with ε fixed at 0.85, while varying the competition parameter c_N_ = 0, 0.04, and 0.37. Figures 5A and 5B show that the model was sensitive to c_N_; a higher c_N_ implies a lower replacement of VT by NVT after the introduction of the vaccine; therefore, the VT incidences are higher and the NVT incidences are lower compared with a lower c_N_ in all age groups. In Figure 5C, the overall incidence is about the same for all three parameters. However, some effects of the different values of c_N_ were apparent once we stratified by VT or NVT incidences. Here, when c_N_ = 0.37, the overall IPD incidence decreased in the age groups 0–1 and 45–64 years, was constant in age groups 2–44 years, and increased for people aged ≥65 years. This is because there was a combination of two opposing effects: increase of VT incidence and decrease of NVT incidence. The model predicted an overall decrease in IPD incidence in the 0–1 years age group. Specifically, the overall IPD incidences (per 100,000 population) 20 years after the introduction of PCV13 in the 0–1 years age group were 118.65 if c_N_ = 0, 118.00 if c_N_ = 0.04, and 105.43 if c_N_ = 0.37. However, the overall IPD incidences (per 100,000 population) 20 years after the introduction of the vaccine in the ≥75 years age group were 491.94 if c_N_ = 0, 492.44 if c_N_ = 0.04, and 498.22 if c_N_ = 0.37.

**Figure 6:**
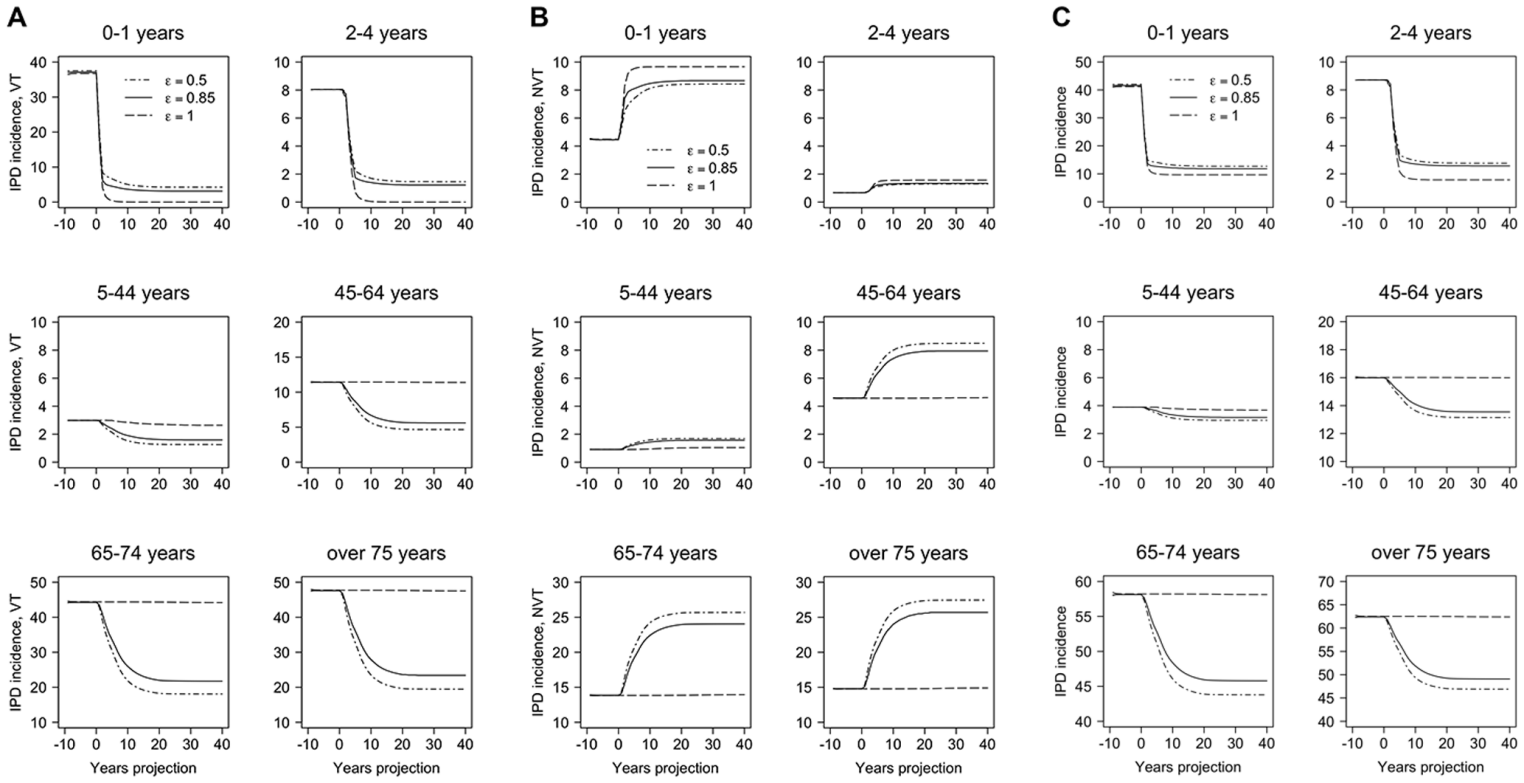
Yearly incidence of IPD with different mixing parameters. **A**, IPD incidence, VT; **B**, IPD incidence, NVT; and **C**, IPD overall incidence. Yearly incidence per 100,000 population over time when different mixing parameters are used: ε = 0.50, 0.85, 1.00; and the competition parameter is c_N_ = 0.04. Each panel corresponds to a different age group. Note: the y-axis scales are different on each graph. Abbreviations: c_N_, competition parameter; ε, mixing paremeter; IPD, invasive pneumococcal disease; NVT, non-vaccine types; VT, vaccine types.

**Figure 6** presents VT, NVT, and overall IPD incidence per 100,000 population, over time with c_N_ fixed at 0.04, while varying the mixing parameter ε = 0.50, 0.85, and 1.00. As higher values of ε imply less mixing, IPD incidence, therefore, had a larger decrease over time when the mixing was more proportionate (ε = 0.50) leading to a higher herd protective effect. The opposite happened when mixing was fully assortative (ε = 1.00), and incidence remained nearly unchanged for older unvaccinated age groups.

## Discussion

Using available Dutch data and a transmission dynamic model, we estimated that 10 years after the introduction of PCV13, the number of IPD cases per year (both VT and NVT) for children <2 years of age would be 45, reduced from 160 cases per year if no vaccination had ever been implemented. At 10 years the equilibrium was not fully reached yet; hence, after 30–40 years, the IPD cases per year for children <2 years of age reached 44. For the elderly ≥75 years of age, IPD cases per year fell from 652 to 573 at equilibrium around 30 years after PCV13 introduction. Overall, IPD cases per year in the Netherlands were predicted to decrease from 2623 cases per year pre-vaccination to 2475, 2289, 2185, 2179, and 2178 cases per year at 5, 10, 20, 30, and 40 years post-vaccination. Therefore, vaccination with PCV13 in the Netherlands was predicted to lower IPD cases annually by up to 445 cases per year in the medium- to long-term.

Rozenbaum *et al* (2010) [41] recently estimated the cost-effectiveness of PCV13 compared with no vaccination using a static model that included herd protection and serotype replacement in a straightforward linear relationship (termed “net indirect effects”). Using a total vaccination cost of approximately €300 for 4 doses, they estimated cost-effectiveness at approximately €50,000 per quality-adjusted life year, based on a similar reduction in IPD-cases in children aged ≤5 years, as found in this study. However, the linear “net indirect effects” calculus indicated a further reduction of only 181 cases; i.e. a total reduction of 331 cases per year. This is in contrast to our current analysis based on herd protection and serotype replacement within a population dynamic design. Notably, here we estimated a reduction in IPD cases already beyond this number after 10 years, increasing to a reduction of approximately 440 cases per year 20–40 years post-vaccination. Although we acknowledge that our comparison was hampered by the different designs of both models, we do note that both were built on the same dataset for IPD cases. Therefore, a suggestion of the change in cost-effectiveness might have some validity. Inserting the outcomes of the dynamic model developed here into the cost-effectiveness model of Rozenbaum *et al* (2010) [41] would improve the cost-effectiveness profile of PCV13. Note that this estimate only concerns the reduction in IPD cases; thus, potential further benefits in otitis media and pneumonia might further improve cost-effectiveness.

Our results were quite robust for the sensitivity analyses that we performed on the parameters that regulate herd immunity (ε) and competition (c_N_). Using PCV7 data, ε was estimated at 0.85, which is quite high, considering that 1.00 reflects full assortativeness. Variation over the range 0.50–1.00 did not influence results dramatically. However, a slight trend could be seen where lower values of ε (more proportionate mixing pattern) facilitated elimination of VT-transmission with correspondingly higher reductions in IPD cases. Furthermore, increasing the value of the competition parameter c_N_ — reflecting lower protection from VT carriage — generally resulted in lower replacement of VT by NVT after introduction of vaccination, and thus resulted in fewer IPD cases, whereas a lower c_N_ showed the opposite effect. However, for the older age groups, the trend may reverse and thus stabilise the overall number of IPD cases, explaining why the results are relatively insensitive to the competition parameter.

As with all models, our model is limited by being a simplification of reality and thus the above considerations should be taken with caution. Our current model consisted of simplified population structures and dynamics involving 100 cohorts of individuals (0, 1, 2, 3…99) each corresponding to one year of age and each of equal size, with a total stable population (i.e. births equal deaths). An additional crucial point of the current work relies on model parameter estimates which, due to the lack of data from the Netherlands, had to be extrapolated from other countries. In particular, the competition parameter between VT and NVT serotypes was based on estimates derived for England and Wales, where post-PCV7 data were available for several years [32]. Vaccine efficacy against VT colonisation and duration of protection were also taken from a study in England and Wales, and were 52% and 5 years, respectively [40]. Although this is clearly limiting the validity of our results, to our knowledge, these represent the best sources of information concerning competition between serotypes, as well as efficacy parameters being derived using pre- and post-vaccination IPD data from the England and Wales surveillance system. Finally, no natural immunity was included in the model because the generation of naturally-acquired immunity to pneumococcal serotypes is too complex and poorly understood to be incorporated in the present model structure. Clearly, all these assumptions can be updated when additional data become available. This also applies to any desired transitions from lower-valent vaccines to PCV13 as suggested by our and potentially other analyses. For example, there are no data yet on the transition in the first year of life from PCV7 to PCV13. Long-term surveillance of IPD, carriage, and non-bacteraemic pneumonia are crucial to understand vaccine effects over the long term.

Another limitation is that this model only captures invasive disease. Efficacy and effectiveness studies of PCV7 and PCV13 have shown a substantial impact on non-invasive disease as well. These disease states are not included in our analysis because serotype-specific data on pneumonia and acute otitis media are too scarce to attempt to predict long-term effects of vaccine pressures on VT and NVT. Again, further surveillance studies on non-IPD will be crucial in evaluating the further impact the vaccine will have on these conditions.

Currently in the Netherlands PCV10 is used having replaced PCV7 in 2011. To date, the impact of PCV10 on carriage remains uncertain [42]–[45], and a herd effect has not yet been demonstrated. For this reason PCV10 was not included in the transmission dynamic analysis. If and when such data become available, the analysis can be updated to include the effects of PCV10.

Miller *et al* (2011) [46] have shown that the incidence of IPD caused by PCV13-only serotypes halved in children aged <2 years in England and Wales where PCV13 replaced PCV7 in 2010. This is due to protection against serotypes 7F and 19A in particular, which were the main causes of replacement disease. However, Miller *et al* (2011) [46] claimed that it was too early to see indirect effects in older ages or serotype replacement effects in the vaccinated population. However, in the US, indirect effects have been observed with no evidence yet of serotype replacement in all adult age groups within 1.5 years of implementation of the PCV13 National Immunisation Programme [47], [48].

In conclusion, we have adapted to the Netherlands setting the first application of transmission dynamic modelling for the spread of pneumococci and potential subsequent IPD. We estimated a base-case reduction in IPD due to PCV13 vaccination of up to 445 cases per year, including herd protective and serotype replacement indirect effects. When compared with the previously-published static model [41], which included a linear approximation of indirect effects, we estimated slightly higher reductions in IPD in the Netherlands due to these indirect effects, potentially translating to more favourable cost-effectiveness of PCV13 than previously indicated. Sensitivity analyses showed that results are sensitive to the competition parameter arranging the exchange between VT and NVT, and the type of population mixing. This work is the first attempt to quantify the overall effects of a PCV13 programme in the Dutch population taking into account all the direct and indirect effects on IPD. However, further research is clearly needed in order to evaluate the potential overall effects on non-IPD, which represent a major widespread burden in terms of both morbidity and cost.
